# Transcriptomics and metabolomics reveal hypothalamic metabolic characteristics and key genes after subarachnoid hemorrhage in rats

**DOI:** 10.1007/s11011-024-01363-2

**Published:** 2024-06-06

**Authors:** Zongchi Liu, Zhaohui Chai, Fan Wu, Luyuan Zhang, Xiaoyi Wang, Zihan Xu, Yuxiang Weng, Jiangbiao Gong, Jian Shen, Renya Zhan, Yu Zhu

**Affiliations:** Department of Neurosurgery, First Affiliated Hospital, College of Medicine, Zhejiang University, Hangzhou, 310000 China

**Keywords:** Subarachnoid hemorrhage, Hypothalamus, Transcriptomics, Metabolomics

## Abstract

Subarachnoid hemorrhage (SAH) is a serious hemorrhagic event with high mortality and morbidity. Multiple injurious events produced by SAH can lead to a series of pathophysiologic processes in the hypothalamus that can severely impact patients’ life. These pathophysiologic processes usually result in physiologic derangements and dysfunction of the brain and multiple organs. This dysfunction involved multiple dimensions of the genome and metabolome. In our study, we induced the SAH model in rats to obtain hypothalamic tissue and serum. The samples were subsequently analyzed by transcriptomics and metabolomics. Next, the functional enrichment analysis of the differentially expressed genes and metabolites were performed by GO and KEGG pathway analysis. Through transcriptomic analysis of hypothalamus samples, 263 up-regulated differential genes, and 207 down-regulated differential genes were identified in SAH groups compared to Sham groups. In the KEGG pathway analysis, a large number of differential genes were found to be enriched in IL-17 signaling pathway, PI3K-Akt signaling pathway, and bile secretion. Liquid chromatography-mass spectrometry metabolomics technology was conducted on the serum of SAH rats and identified 11 up-regulated and 26 down-regulated metabolites in positive ion model, and 1 up-regulated and 10 down-regulated metabolites in negative ion model. KEGG pathways analysis showed that differentially expressed metabolites were mainly enriched in pathways of bile secretion and primary bile acid biosynthesis. We systematically depicted the neuro- and metabolism-related biomolecular changes occurring in the hypothalamus after SAH by performing transcriptomics and metabolomics studies. These biomolecular changes may provide new insights into hypothalamus-induced metabolic changes and gene expression after SAH.

**Supplementary Information:**

The online version contains supplementary material available at 10.1007/s11011-024-01363-2.

## Introduction

Subarachnoid hemorrhage (SAH) is a devastating disease with a high mortality rate of 22–45%, accounting for 3% of all strokes (Kaplan et al. 2023; Chai et al. 2023). 80% of SAH cases are caused by rupture of intracranial aneurysm, and other causes include vascular malformation and vasculitis (Lawton and Vates 2017). The fundamental pathology in SAH is the extravasation of blood circulation products in the central nervous system into the subarachnoid space (Hall and O’Kane 2018). Due to serious hemorrhagic events leading to the strong stimulation of intracranial vessels and tissues by blood by-products, cause a series of the brain–organ interactions including cardiovascular, respiratory, endocrine, hematological, and immune disorders (Stevens and Nyquist 2007). The blood entering into the subarachnoid space can directly generate mechanical pressure on the hypothalamus and lead to local ischemia in this region due to the increase of intracranial pressure (Hasegawa et al. 2022). Oxidative stress, inflammation, glial activation, blood-brain barrier (BBB) disruption, and apoptosis also lead to SAH-related Regional ischemia (Macdonald 2014; Ma et al. 2018). These events led to the release of higher levels of glutamine from the hypothalamus (Takemoto et al. 2020), the activation of the sympathetic spirit by controlling the HPA axis (hypothalamus-pituitary-adrenal axis) (Chen et al. 2017), and the release of Vasopressin into the blood circulation (Aleksandrowicz and Kozniewska 2022), resulting in a series of pathophysiological processes.

Multi-omics analysis technologies are increasingly used to clarify the pathogenesis and potential pathogenic changes related to diseases (Zhao et al. 2020). At present, transcriptome technology has been employed to determined genes differentially expressed in the whole bilateral hippocampus remote from the SAH bleeding site and provided the first systematic gene and pathway database of the hippocampal response after SAH (Regnier-Golanov et al. 2021). In another example, transcriptome analysis identified differentially expressed genes in SAH-induced microglia, indicating that microglia are rapidly activated after SAH and mediate the regulation of inflammation (Xu et al. 2021). It has been found that SAH can change cerebrospinal fluid (CSF) metabolites involved in carbohydrate, lipid, and amino acid metabolism through metabolomics research on cerebrospinal fluid (Li et al. 2019b). The current review showed that metabolomics well describes the biochemical changes in post-hemorrhage CSF, with the ability to detect ischemic markers such as lactate, pyruvate, glucose, and glutamate (Ho et al. 2023). Detection of changes in energy-supplying molecules in CSF after SAH can visualize the suffering of neural and glial cell damage and strongly suggest a prognosis (Lasica et al. 2023).

To our knowledge, there are no transcriptomic and metabolomic analyses addressing hypothalamic changes after SAH. Moreover, the molecular-level biological mechanisms affected by the hypothalamus after subarachnoid hemorrhage are still not completely clear. Thus, integrative analysis of transcriptomics and metabolomics may contribute to a deep understanding of the metabolic mechanisms between the hypothalamus and metabolites. Our data revealed the relationship between the hypothalamus and bile acid metabolism for the first time and may provide useful information into the study of the metabolic characteristics of the hypothalamus after subarachnoid hemorrhage.

## Methods

### Animal experiment

Animal procedures were licensed by the Ethics Committee of the First Affiliated Hospital of Zhejiang University and were performed by the Laboratory Animal Care and Guidelines. Animals were kept in an animal institution according to a 12-hour light/night cycle with ad libitum access to food and water. Eight male SD rats of approximately 12 weeks of age from the same batch were selected for the study and subsequently randomized into two groups: SAH (*n* = 4) and Sham (*n* = 4). Experimental SAH was caused by perforation of the Willis ring (Anzabi et al. 2018). In detail, anesthesia was induced in a room filled with isoflurane, and the rats were subsequently fixed on the stereotaxic apparatus holder. The skin around the neck was sterilized using alcohol-soaked cotton balls. Under sterile conditions, subcutaneous structures were sequentially exposed through a midline neck incision. The common carotid artery, internal carotid artery, and external carotid artery were detected. Three ligature wires were used to place under the three vessels and the external carotid artery was severed between the two ligature wires. A single-stranded wire was then inserted through the stump into the internal carotid artery and moved forward. Slight resistance was detected and the entry was continued for 1 mm, thus puncturing the bifurcation of the middle cerebral and anterior cerebral arteries. The wound was closed by pulling out the thin wire and ligating the stump. In the Sham group of rats, the monofilament was withdrawn without puncturing the vessel after being pushed to the point of resistance. The rats were tested regularly every day for 96 h after surgery.

### RNA extraction and RNA sequencing

Rats were sacrificed by euthanizing with carbon dioxide on the fourth day after surgery, followed by thoracotomy and decapitation. The SAH and Sham groups were immediately subjected to hypothalamus collection by double-blind method by the experimenter. Samples were verified for RNA integrity and the presence of DNA contamination by agarose gel electrophoresis according to the manufacturer’s instructions. RNA purity (OD260/280 and OD260/230 ratios) was detected by a NanoPhotometer spectrophotometer (IMPLEN, CA, USA) and RNA integrity was further assessed using the RNA Nano 6000 assay kit for the Bioanalyzer 2100 system (Agilent Technologies, CA, USA). All RNA-seq libraries were constructed using the NEBNext® UltraTM RNA Library Prep Kit for Illumina® (NEB, USA) kit, and library quality was assessed on an Agilent Bioanalyzer 2100 system. After the libraries were qualified, they were sequenced on the Illumina Novaseq platform and 150 bp paired-end reads were generated.

### RNA sequencing analysis

In order to ensure the quality and reliability of data analysis, the raw data were filtered, and all the downstream analyses were based on high-quality clean data with high quality. Criteria mainly included removing reads with adapters, N-containing reads (N means uncertain base information), and low-quality reads (reads with Qphred ≤ 20 bases accounting for more than 50% of the total read length). In addition, Q20, Q30, GC content, and sequence duplication levels were calculated to ensure the data quality. The index of the reference genome was constructed with HISAT2 (v2.0.5), and paired-end clean reads were aligned to the reference genome via HISAT2 (v2.0.5). We selected HISAT2 as the comparison tool for that HISAT2 can generate a database of splice junctions based on gene model annotation files and, thus, offer a better alignment effect than other non-splice comparison tools. The mapped reads of each sample were assembled by StringTie (v1.3.3b) and the novel transcripts prediction was performed.

FeatureCounts (v1.5.0-p3) was then used to count the reads numbers mapped to each gene. The fragments per kilobase million (FPKM, expected number of fragments per kilobase of transcript sequence per millions base pairs sequenced) of each gene were calculated on the basis of the gene length and reads count mapped to this gene.

Differential expression analysis between the two combinations of comparison was performed using DESeq2 software (1.16.1). Genes found to have an adjusted *P* value (a common form of false discovery rate) < 0.05 by DESeq2 were assigned as differentially expressed. *P* values were adjusted using the Benjamini & Hochberg method. The corrected *P* value and |log2foldchange| were used as thresholds for significant differential expression.

#### Enrichment analysis of the differential expressed genes

Gene Ontology (GO) and Kyoto Encyclopedia of Genes and Genomes (KEGG) enrichment analysis were performed on the common DEGs using the clusterProfiler package in R software. GO terms and KEGG pathways with adjusted *p*-values less than 0.05 were considered to be significantly enriched by differentially expressed genes.

### Liquid chromatography-mass spectrometry (LC-MS) analysis

Liquid chromatography-mass spectrometry (LC-MS)-based untargeted metabolomics analysis and data processing were performed to analyze the metabolites in serum of SAH groups and Sham groups (ultra-high performance liquid chromatography (UHPLC) coupled with high-resolution mass spectrometry). UHPLC-MS/MS analyses were performed using a Vanquish UHPLC system (Thermo Fisher, Germany) coupled with an Orbitrap Q Exactive TMHF-X mass spectrometer (Thermo Fisher, Germany) in Novogene Co., Ltd. (Beijing, China). Serum samples were injected onto a Hypesil Gold column (100 × 2.1 mm, 1.9 μm) using a 12-min linear gradient at a flow rate of 0.2mL/min. Eluent A (0.1% FA in Water) and eluent B (Methanol) were used as the eluents for the positive polarity mode. Similarly, the eluents for the negative polarity mode were eluent A (5 mM ammonium acetate, pH 9.0) and eluent B (Methanol). Solvents were applied with the following gradient: 2% B, 1.5 min; 2–85% B, 3 min; 85–100% B, 10 min; 100-2% B, 10.1 min; 2% B, 12 min. The Q ExactiveTM HF-X mass spectrometer was operated in full-scan and polarity-switching mode with spray voltage of 3.5 kV, the capillary temperature of 320 °C, sheath gas flow rate of 35 psi, auxiliary gas flow rate of 10 L/min, S-lens RF level settled at 60, and auxiliary gas heater held at 350 °C.

The raw data files generated by UHPLC-MS/MS were processed using Compound Discoverer 3.1 (CD3.1, Thermo Fisher) to perform peak alignment, peak picking, and quantitation for each metabolite. Subsequently, the total spectral intensity was used to normalize peak intensities. Additive ions, molecular ion peaks, and fragment ions were predicted on the basis of normalized data. In order to obtain accurate qualitative and relative quantitative results, peaks were matched with the mzCloud (https://www.mzcloud.org/), mzVault, and MassList databases.

Utilized the KEGG database (https://www.genome.jp/kegg/pathway.html), HMDB database (https://hmdb.ca/metabolites), and Lipidmaps database (http://www.lipidmaps.org/) to annotate the metabolites. Principal component analysis (PCA) and partial least squares discriminant analysis (PLS-DA) were performed at metaX. Metabolites with VIP > 1, *P*-value < 0.05, and fold change (FC) ≥ 2 or FC ≤ 0.5 were considered to be differential metabolites.

## Results

### Transcriptomic analysis revealed differentially expressed genes and pathways between SAH and control samples

**Fig. 1:**
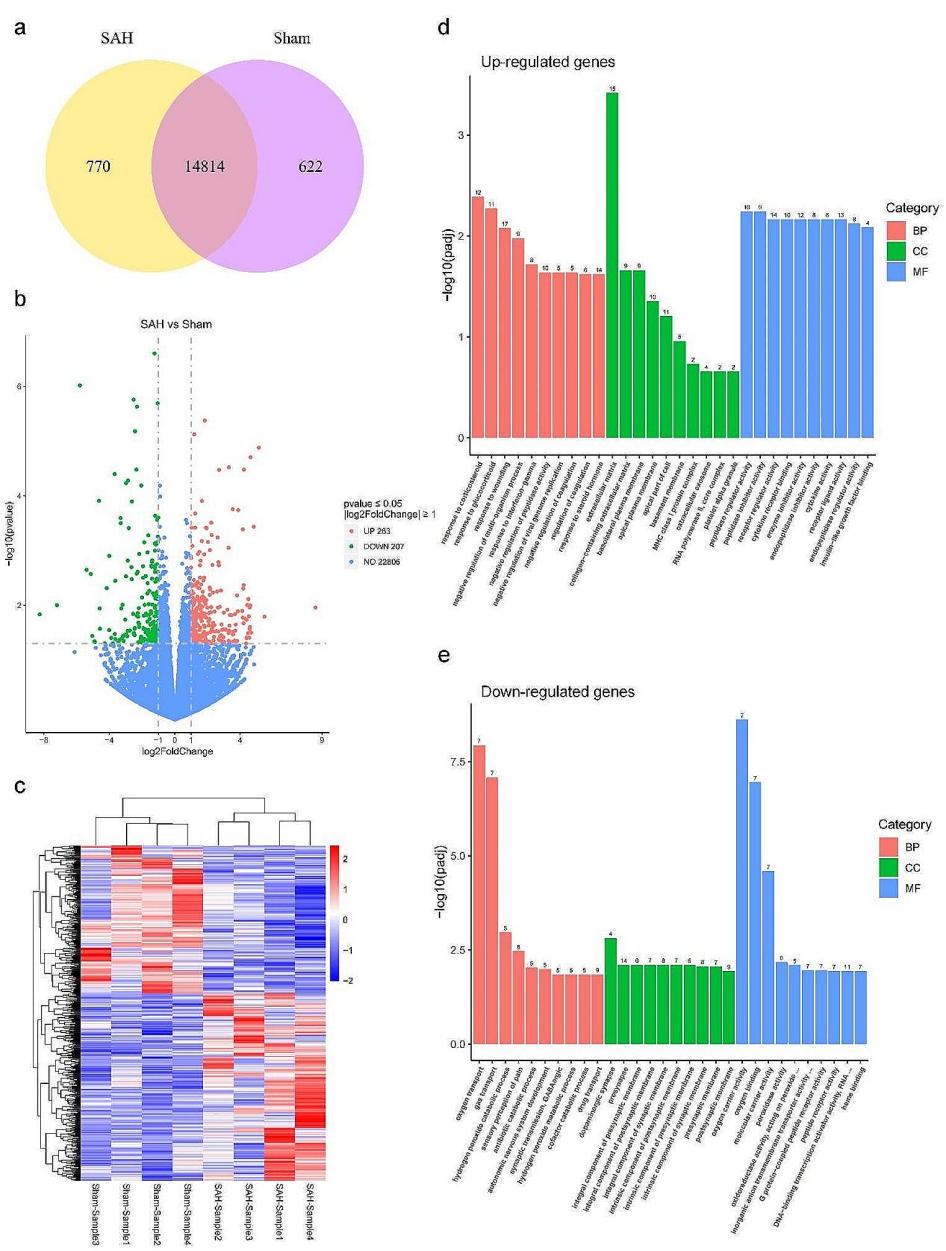
Profiling of different expression genes (DEGs) in rats between SAH groups and Sham groups. **a** Venn diagram showing the number of genes in SAH groups and Sham groups. **b** Volcano plot of RNA-seq results showing statistics of up-regulated and down-regulated genes (log2FC ≥ 1; *p*-value ≤ 0.05). **c** Heatmap of DEGs in SAH groups compared with Sham groups. **d** Go enrichment analysis of up-regulated genes **e** Go enrichment analysis of down-regulated genes. BP: Biological process; CC: Cellular component; MF: Molecular function

Genes with FPKM value greater than 1 in the transcriptomics data were considered as expressed genes. Venn diagram demonstrated the distribution of the number of genes annotated only in SAH group (770), only in Sham group (622), or in both groups (14,814) (Fig. 1a). Transcriptional changes in SAH were determined and the screening conditions for differentially expressed genes (DEGs) were |Log2FoldChange| ≥ 1 and Padj ≤ 0.05. Therefore, the distribution of 263 up-regulated differential expressed genes and 207 down-regulated differential expressed genes was visualized more by volcano plots (Fig. 1b). DEGs were clustered in a heatmap to highlight transcriptomic differences between SAH groups and Sham groups (Fig. 1c). To determine relevant biological processes after SAH, we analyzed the top 20 differential genes by GO enrichment analysis (Supplementary Fig. 1). We found that the differential genes in biological processes (BP) were mainly involved in “gas transport” and “oxygen transport”. Cellular Component (CC) showed that differential genes are heavily clustered in the extracellular matrix and exert functions such as oxygen carrier activity, oxygen binding, and molecular carrier activity. We further performed GO analysis on up-regulated genes and down-regulated genes separately (Fig. 1d, e). Based on the results we found that the up-regulated genes were more concentrated in “response to corticosteroid”, “response to glucocorticoid”, and “response to wounding”, and were significantly enriched in the extracellular matrix, while the down-regulated genes were mainly enriched in “gas transport” and “oxygen transport”. The top 5 genes related to the above specific GO terms were shown in Supplementary Table 1.

### KEGG pathway analysis reveals neuro-related signaling and metabolism-related signaling

**Fig. 2:**
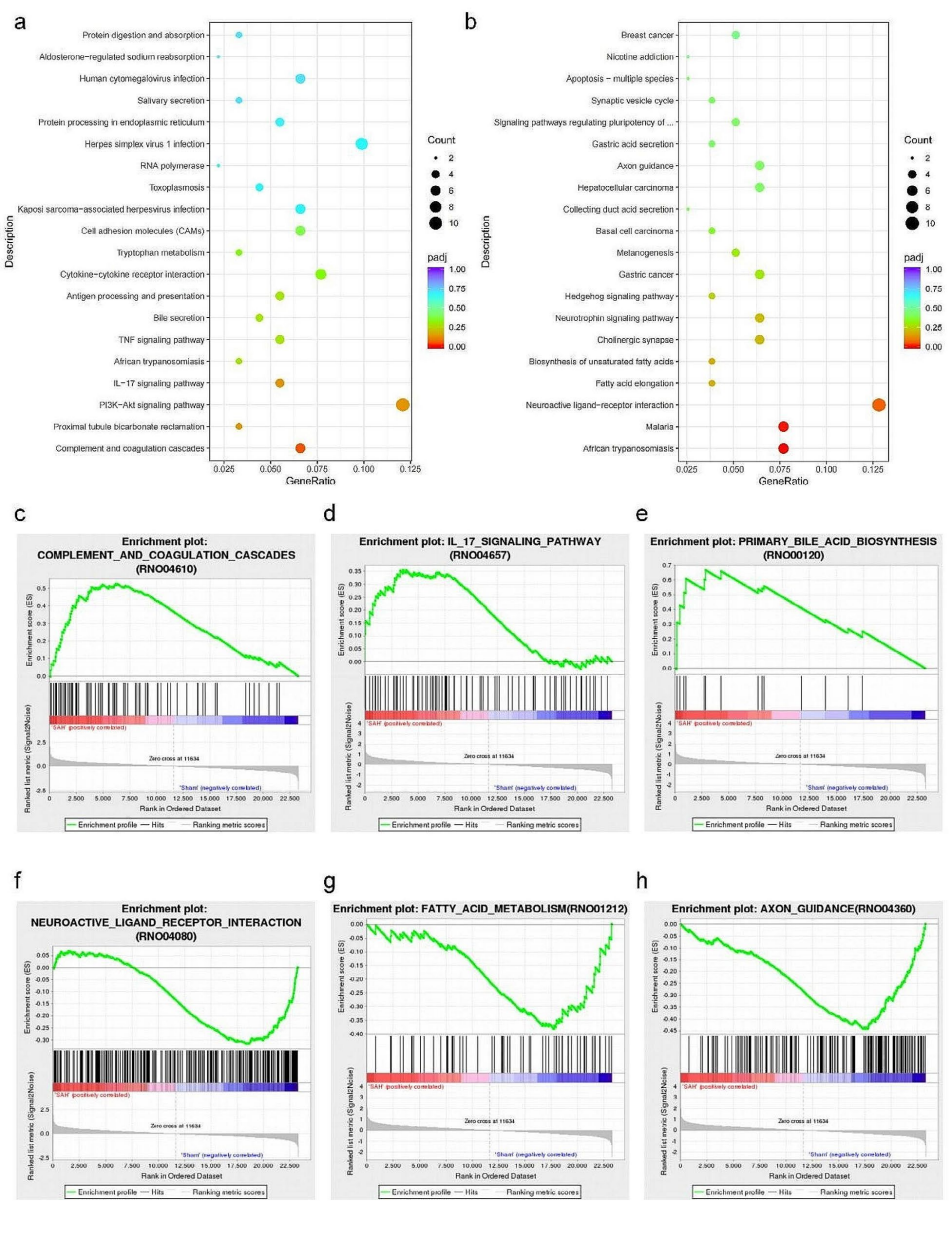
The functional enrichment analysis of DEGs. **a** and **b** Scatter plot of the top 20 KEGG pathways enriched among the up-regulated and down-regulated genes, respectively. **c** to **h**. Gene set enrichment analysis (GSEA) showing neuro-related signaling, immune-related signaling, and metabolism-related signaling

**Fig. 3:**
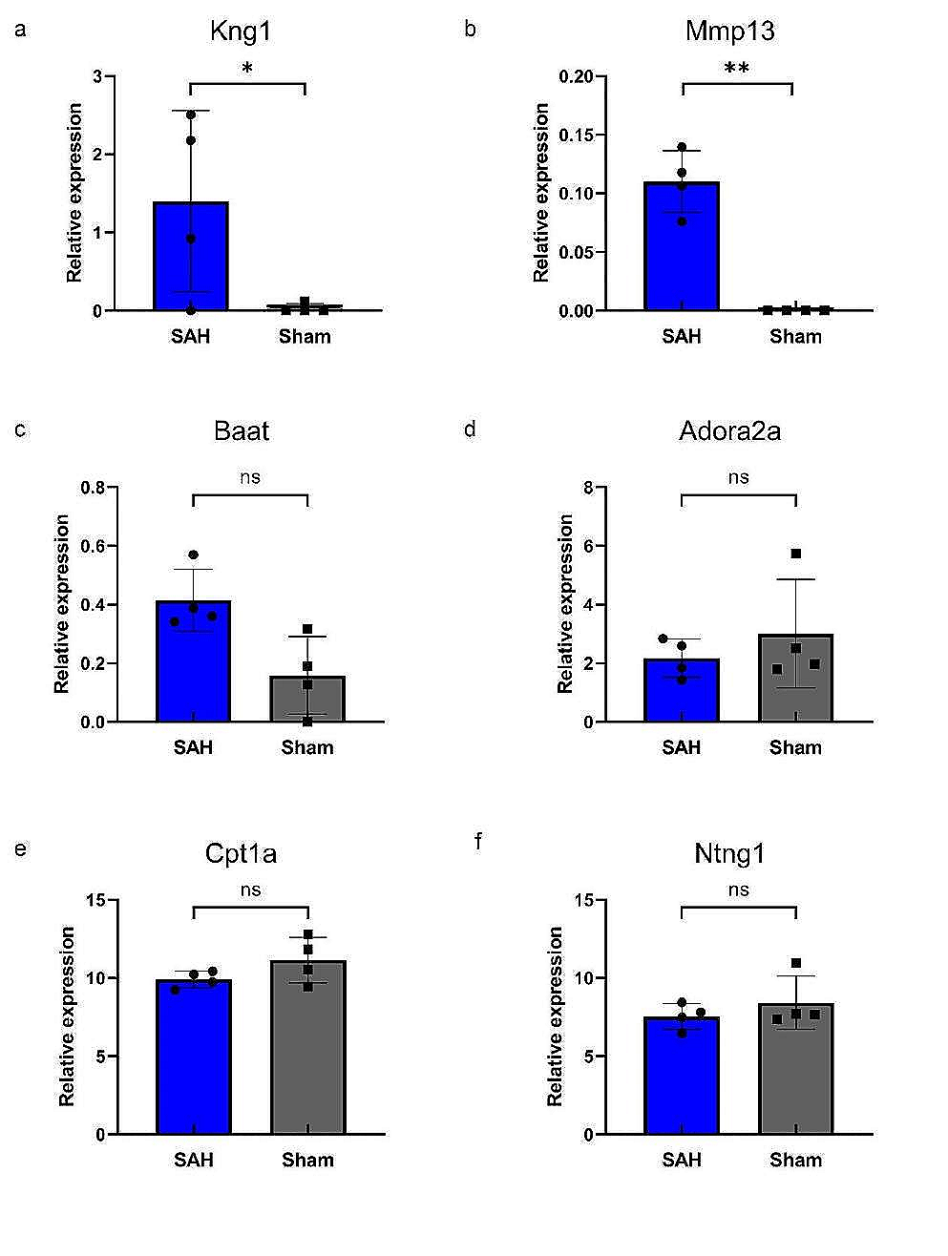
Comparison of the gene expression of RNA-seq. The data represent the relative gene expression using FPKM value of **a** Kng1, *P* = 0.016, **b** Mmp13, *P* = 0.009, **c** Baat, *P* = 0.110, **d** Adora2a, *P* = 0.290, **e** Cpt1a, *P* = 0.327, **f** Ntng1, *P* = 0.458. *P < 0.05; **P < 0.01; ***P < 0.0001. *N* = 8, with *n* = 4 for SAH, *n* = 4 for Sham

KEGG enrichment analysis was carried out for the above DEGs, and the top 20 significantly enriched pathways were shown on bubble charts (Fig. 2a, b). We found that up-regulated genes were associated with complement and coagulation cascades, IL − 17 signaling pathway, PI3K-Akt signaling pathway, and bile secretion, while down-regulated genes were associated with African trypanosomiasis, malaria, neuro-related signaling (Neuroactive ligand-receptor interaction and Axon guidance), and metabolism-related signaling (Fatty acid elongation). Additional validation of the expression of these pathways was performed using GSEA (Fig. 2c to h and Supplementary Table 2). The top five most up-regulated genes related to immunity include: Kng1, Procr, LOC100911545, coagulation factor III (F3), and coagulation factor II (F2). The top five most up-regulated genes related to the IL-17 signaling pathway include: Mmp13, Mmp3, Nfkbia, Hsp90aa1, and Ikbkb. The top five most up-regulated genes related to primary bile acid biosynthesis include: Baat, Cyp27a1, Cyp39a1, Acox2, and Hsd3b7. The top five most down-regulated genes associated with Neuroactive ligand-receptor interaction include: Adora2a, Gria3, Ptger4, Tacr3, and Thra. The top five most down-regulated genes associated with fatty acid metabolism include: Cpt1a, Elovl5, RGD1560015, Ehhadh, and AABR07039037.1. The top five most down-regulated genes associated with axon guidance include: Ntng1, Arhgef12, Fyn, LOC100910732, and Efna3.Then we further analyzed the expression levels of the top expressed genes in different groups by scatter bar chart (Fig. 3a to f). We found that Kng1 and Mmp13 were significantly different in the SAH and Sham groups.

### Metabolites profiling of SAH showed significant difference compared with sham group

**Fig. 4:**
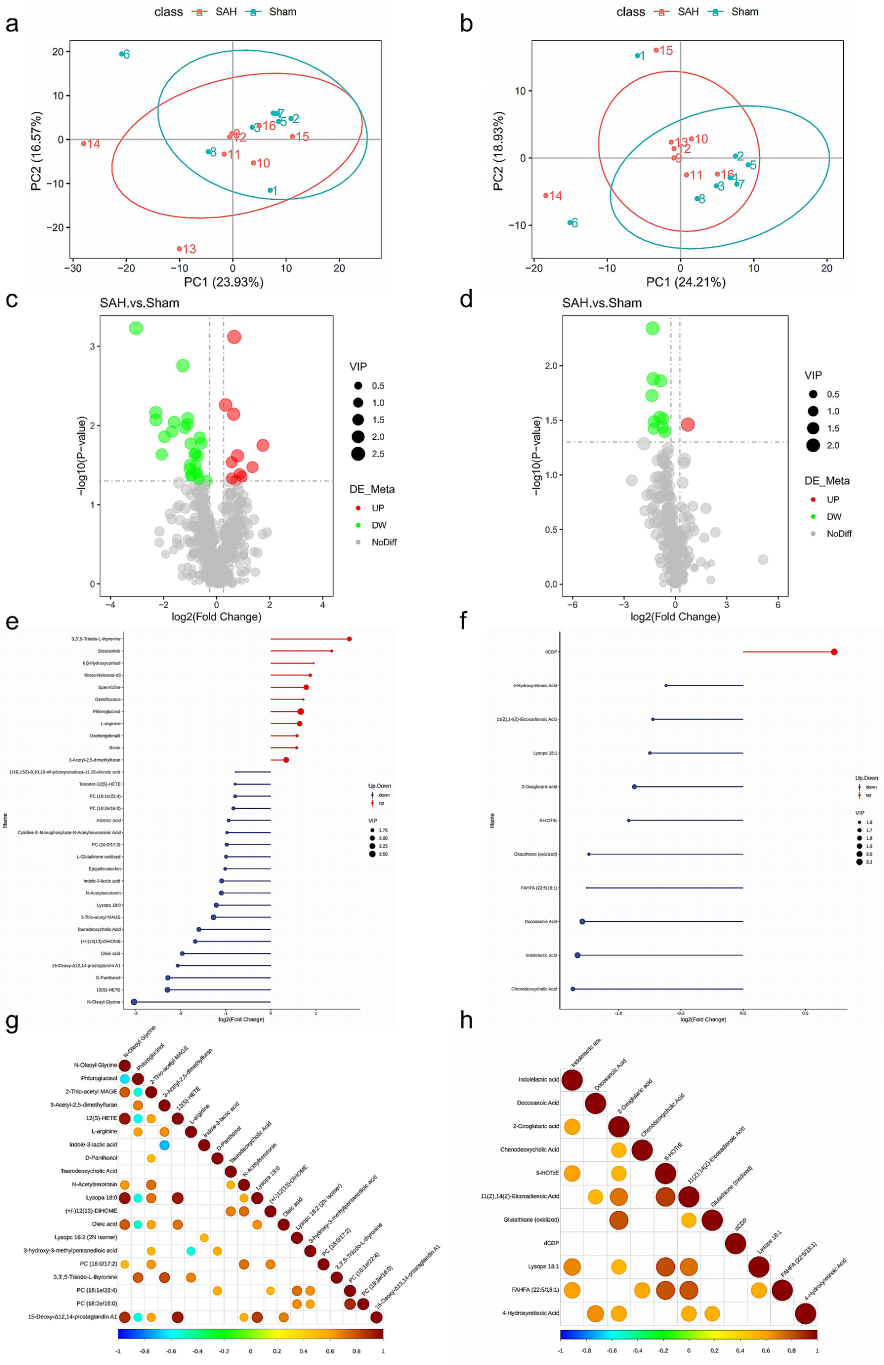
Analysis of differentially expressed metabolites (DEMs) between SAH groups and Sham groups. **a** and **b** PCA was performed for dimension reduction analysis of metabolites in rats. Axes showed the percentage of variance of the first two components (PC1, PC2). The ellipse was the 95% confidence interval. **c** and **d** Volcano plot of the significant differences in the DEMs. The horizontal and vertical axes represent the fold-change (FC) and *P*-value, respectively. VIP: Variable important in projection. **e** and **f** Matchstick chart showed the top 20 up- and down-regulated metabolites. The color of the point represented up- and down-regulation. Red represented up-regulation, and blue represented down-regulation; the length of the bar represented the value of log2 (FoldChange); the size of the point represented the VIP value. **g** and **h** Pearson correlation coefficient to analyze the correlation between various metabolites. The positive correlation tended to be 1 and the negative correlation tended to be -1. All left figures: positive ion model; All right figures: negative ion model

Based on the transcriptome results, we performed metabolomic characterization against sera from SAH rats and Sham rats. The PCA results of all samples in positive and negative ion mode were demonstrated in Fig. 4a, b. The samples from the SAH and Sham groups were clearly separated, exhibiting significant differences between the two groups. Partial Least Squares Discrimination Analysis (PLS-DA) was a supervised statistical method for discriminant analysis, and the relationship between metabolite expression and SAH samples was modeled using partial least squares regression. PLS-DA analysis showed that the samples were significantly separated into the SAH and Sham groups (Supplemental Fig. 2, a and b). Moreover, the relative levels of the metabolites were measured at the same level based on z-scores (Supplementary Fig. 2, c and d), Via LC-MS metabolomics analysis, 779 annotated metabolites, including 526 positive ion mode metabolites and 253 negative ion mode metabolites, were identified. Among the above 779 annotated metabolites, 48 metabolites were differentially expressed metabolites (DEMs). Further screening of 48 DEMs, we identified 11 up-regulated and 26 down-regulated metabolites in positive ion model, and 1 up-regulated and 10 down-regulated metabolites in negative ion model (Fig. 4c, d). According to the results of the quantitative analysis, to visualize the differential metabolites, we further demonstrated the up- and down-regulation of differential metabolites and metabolites with large fold-change in differences (Fig. 4e, f). We found that 3,3’,5-Triiodo-L-thyronine was the most up-regulated metabolite and N-Oleoyl Glycine was the most down-regulated metabolite in the positive ion model. While in the negative ion model, dCDP was the only up-regulated metabolite and chenodeoxycholic acid, indolelactic acid and docosanoic acid showed significant down-regulation. Up- and down-regulated differential metabolites were clustered in a heat map used to highlight metabolomic differences between the SAH and Sham groups (Supplemental Fig. 3, a and b), and the results showed that the two groups varied largely. Pearson correlation analysis revealed significant associations between the TOP 20 differential metabolites (Fig. 4g, h). Among the positive ion model, 12(S)-HETE had a significant positive correlation with N-Oleoyl Glycine (*r* = 0.950, *P* < 0.001) and Lysopa 18:0 was strongly positive correlated with N-Oleoyl Glycine (*r* = 0.953, *P* < 0.001) and 12(S)-HETE (*r* = 0.938, *P* < 0.001), respectively. Among the negative ion model, strong positive correlations existed for Glutathione (oxidized) and 2-Oxoglutaric acid (*r* = 0.797, *P* < 0.001), and 9-HOTrE was positively correlated with 11(Z),14(Z)-Eicosadienoic Acid (*r* = 0.848, *P* < 0.001), Lysope 18:1 (*r* = 0.808, *P* < 0.001), and FAHFA (22:5/18:1) (*r* = 0.793, *P* < 0.001), respectively.

### Potential metabolic pathways associated with bile acid in SAH

**Fig. 5:**
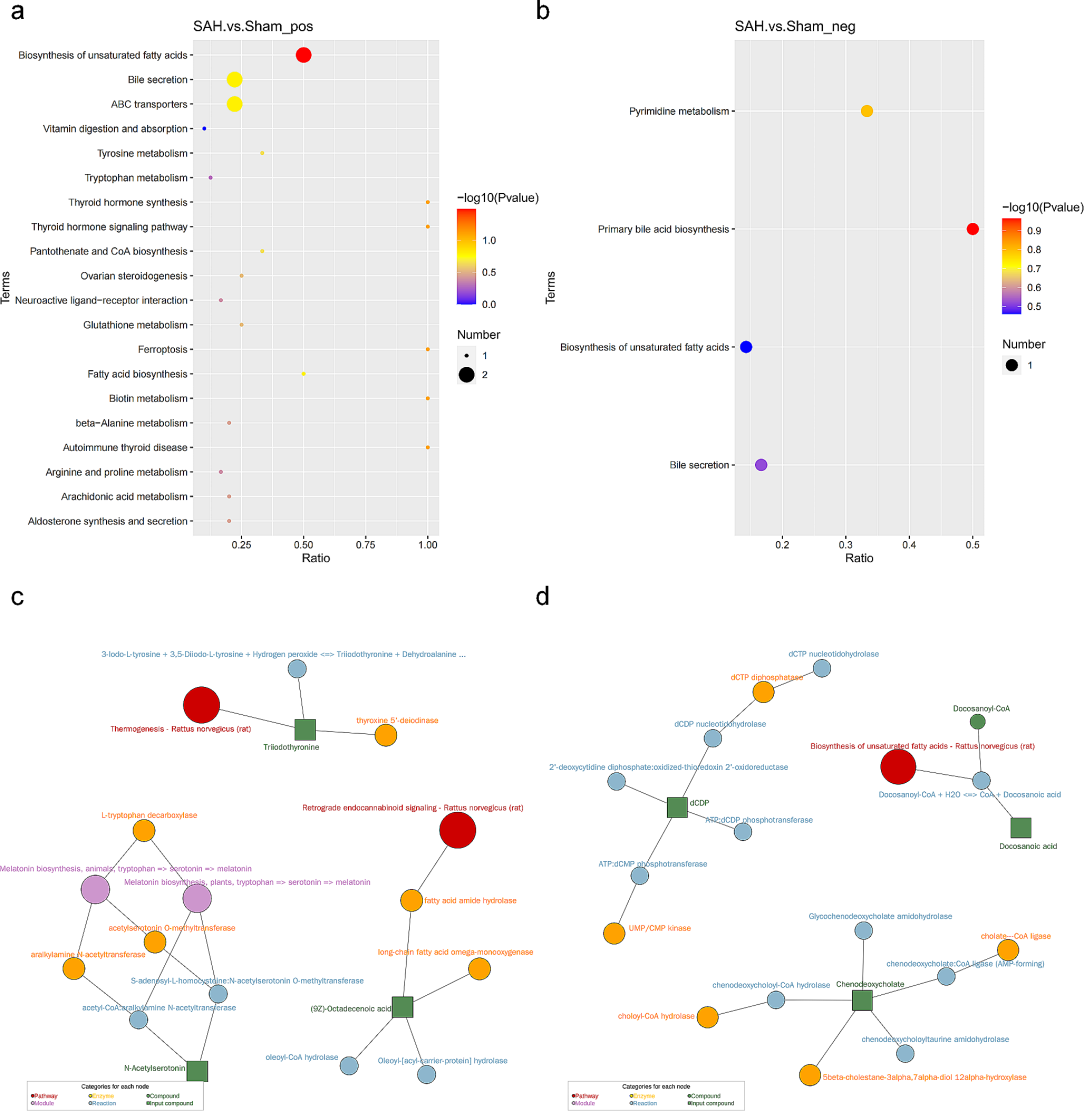
Metabolic pathway analysis of differentially expressed metabolites (DEMs). KEGG enrichment bubble charts of differential metabolites in positive ion model (**a**) and negative ion model (**b**). Network diagram of KEGG enrichment analysis of the top 20 differential metabolites in positive ion model (**c**) and negative ion model (**d**)

As shown in Fig. 5a and b, KEGG pathway analysis showed that DEMs in positive ion model were mainly enriched in pathways such as biosynthesis of unsaturated fatty acids, bile secretion, and ABC transporters. While DEMs in negative ion model were mainly enriched in the pathways of primary bile acid biosynthesis, pyrimidine metabolism, bile secretion, and biosynthesis of unsaturated fatty acids. These results were consistent with what we observed in the transcriptome analysis. Specifically, we performed pathway searches and regulatory network interaction analyses for Top 20 differential metabolites (Fig. 5c, d). Comparing the two groups, we found 17 pathway differences and 16 substance differences in the positive ion model; whereas 10 pathway differences and 15 substance differences in the negative ion model. In addition, we classified metabolite pathway types in multiple dimensions based on HDBM, KEGG, and LIPIDMAPS databases (Supplementary Fig. 4, a to f).

## Discussion

Subarachnoid hemorrhage is a life-threatening neurological condition with high mortality and morbidity rates (Dayyani et al. 2022). There is evidence that intracranial hypertension caused by subarachnoid hemorrhage can alter the function of the hypothalamus (Tan et al. 2017; Ma et al. 2018). However, there have been no further studies on biological functional mechanisms of the hypothalamus at the molecular level after SAH by a multi-omics approach. In our study, we systematically depicted the neuro-, metabolism-, and immune-related biomolecular (genes, metabolites, etc.) changes occurring in the hypothalamus after subarachnoid hemorrhage by performing transcriptomics and metabolomics studies between the SAH and Sham groups. Through transcriptomics we identified genes with altered expression levels in the hypothalamus after the onset of SAH, which were involved in multiple biological processes such as apoptosis, immunity, and metabolic changes. Through metabolomics we obtained comprehensive information about serum metabolites after SAH, which were involved in multiple metabolic pathways such as energy metabolism and metabolite synthesis. Our study suggested that transcriptomics and metabolomics can provide more comprehensive information about the hypothalamus after SAH occurrence from different perspectives.

Similar to a previous study (Regnier-Golanov et al. 2021), we observed significant changes in neurological, immune, and metabolic related gene levels in the hypothalamus of rats 4 days after SAH. Notably, we observed two signaling pathways: the IL-17 signaling pathway and the PI3K-Akt signaling pathway. To our knowledge, few studies currently delve into the role of the IL-17 signaling pathway in SAH. IL-17 was a pro-inflammatory cytokine that synergized with other inflammatory signals to become an important inflammatory effector and amplified inflammatory response in an inflammatory environment (Amatya et al. 2017). The IL-17 signaling pathway can activate inflammatory transcription factors and induce gene expression through NF-KB and activation of the MAPK pathway (p38, ERK, JNK) (Li et al. 2019a). Several studies have shown that elevated levels of IL-17 are observed in both SAH patients and rats, and it had been hypothesized that IL-17 may be a key mediator of neutrophil recruitment into the meninges (Coulibaly et al. 2020; Moraes et al. 2020). Moreover, IL-17 signaling had been shown to mediate white matter damage and cognitive decline in humans and rats by acting on brain endothelial cells via CXCL5 (Xiao et al. 2022). We therefore considered the IL-17 signaling pathway to be a promising therapeutic target. The PI3K-Akt signaling pathway had gained a great deal of research attention for its critical role in regulating neuroimmune inflammation, reducing apoptosis, and attenuating brain tissue damage after SAH (Jin et al. 2022). For example, some studies had found that Aggf1 and CCR2 activate the PI3K-AKT pathway to inhibit neuroinflammation and neuronal apoptosis in vivo and in vitro to exert neuroprotective effects (Zhu et al. 2018; Tian et al. 2022). In addition, changes in immune-related signaling (complement and coagulation cascades) were found in our study. The innate immune system was acutely activated after SAH, and the complement system was a major component of humoral immunity and a bridge linking innate and adaptive immunity during acute SAH (Zhang et al. 2023). Complement activation may induce excessive synaptic pruning by SAH (complement components C1q and C3 are involved in synaptic removal) and cause brain damage (Van Dijk et al. 2020). Another damage mechanism was mediated by the membrane attack complex formed by the C5b-C9 complex (Chai et al. 2023). Similarly, no “membrane attack complex” was observed in our study, which is the same as the results of another study (Regnier-Golanov et al. 2021). Interestingly, we found alterations in metabolic pathways in KEGG pathway: primary bile acid metabolism and fatty acid elongation. This meant that potential changes in bile acid metabolism and lipid metabolism may be affected by the hypothalamus after SAH. Therefore, SAH groups and Sham groups were selected for further serum metabolomics analysis. Based on our data, we similarly found primary bile acid biosynthesis and biosynthesis of unsaturated fatty acids in our metabolomics results, which was consistent with the transcriptomics data. Anatomically, the hypothalamus contained nuclei with cells that control the autonomic nervous system by regulating sympathetic and parasympathetic outflow to the liver, while the hypothalamus controls the secretion of a variety of hormones connected to the liver (Uyama et al. 2004). After brain injury, the mechanism of bile acid metabolism in the liver and brain changed (Huang et al. 2015), and six hours later, the acute phase response (APR) of the liver and the inflammatory system were activated successively, which led to the imbalance of bile acid homeostasis in the brain (Nizamutdinov et al. 2017). A recent prospective study had also confirmed that traumatic brain injury induces alterations in plasma bile acid metabolism and may lead to a drop in metabolic levels of glycocholic, taurocholic, and glycoursodeoxycholic (Zhu et al. 2023). Additionally, the distribution of bile acid receptors and transporter proteins throughout the brain (especially on immune and glial cells) provided a theoretical basis for the possible regulation of neuronal function by bile acids (Bhargava et al. 2020). A previous study found that increased circulating bile acids alter the tight junction structure of the blood-brain barrier, resulting in compromised integrity of the blood-brain barrier (Quinn et al. 2014). A study had confirmed that altered bile acid profiles after traumatic brain injury lead to gastrointestinal dysfunction due to dysregulation of intestinal microorganisms, which seemed to be a potential cause of gastrointestinal dysfunction in patients with SAH (You et al. 2022). However, other researchers thought that bile acids may play a role in protecting neurological function. Wang et al. found that serum total bile acids levels were negatively correlated with clinical severity and hematoma volume in acute cerebral hemorrhage (Wang et al. 2018). In particular, tauroursodeoxycholic acid (TUDCA) can reduce neuronal apoptosis and improve neurological function after SAH through the TGR5/SIRT3 signaling pathway (Wu et al. 2020). Therefore, extensive research was still needed on whether bile acid metabolism mediated beneficial effects in SAH and other bleeding events.

Our study inevitably manifested several restrictions. First, we selected rat hypothalamus and blood for transcriptome and metabolome studies, and in some cases cerebrospinal fluid was still needed for further studies. Secondly, our choice of 4 days after SAH as the time point for the study failed to characterize well the changes that may occur after 4 days, therefore a dynamic study by choosing longer time points including 24 h, 7 days, 30 days, etc. may yield more information.

In summary, this study demonstrated results of transcriptomic and metabolomic analysis on rats with SAH compared with Sham groups. After SAH occurs, the hypothalamus underwent a series of changes at the molecular level, and these changes were reflected in many aspects such as neurological, immune, and metabolic pathways. Transcriptomics results indicated that differentially expressed genes were enriched in IL-17 signaling pathway, PI3K-Akt signaling pathway, and bile secretion. Metabolomics results showed that differentially expressed metabolites were mainly enriched in bile secretion and bile acid biosynthesis. However, how key genes and metabolites affect these pathways have not been thoroughly investigated, and further studies should focus on the detailed mechanisms of hypothalamic-influenced bile secretion and lipid metabolic pathways after SAH.

### Electronic Supplementary Material

Below is the link to the electronic supplementary material.

Supplementary Material 1 (DOCX 1.58 mb)

## Data Availability

The data that support the findings of this study are available on request from the corresponding authors, upon reasonable request.
