# Supplementary material for: Transcriptomics and metabolomics reveal hypothalamic metabolic characteristics and key genes after subarachnoid hemorrhage in rats
Source: Metab Brain Dis. 2024 Jun 6;39(5):679–90. doi: 10.1007/s11011-024-01363-2 (PMC11233374; doi:10.1007/s11011-024-01363-2)
Supplement: Supplementary file 1 — Supplementary Material 1 (DOCX 1.58 mb) [file 11011_2024_1363_MOESM1_ESM.docx]

**Supplementary Table 1 Top five genes of specific GO terms**

| GO terms | Top 5 up-regulated or down-regulated genes | | | | |
| --- | --- | --- | --- | --- | --- |
| Response to corticosteroid | Aqp1 | Ccn2 | Npas4 | Sult1a1 | Abcg2 |
| Response to glucocorticoid | Aqp1 | Npas4 | Sult1a1 | Abcg2 | Ppargc1b |
| Response to wounding | Alox15 | Procr | Aqp1 | Lox | Tm4sf4 |
| Gas transport | Hba-a1 | Hba-a2 | LOC103694857 | LOC103694855 | Aqp1 |
| Oxygen transport | Hba-a1 | Hba-a2 | LOC103694857 | LOC103694855 | LOC100134871 |

**Supplementary Table 2 Top five genes of specific KEGG pathways (Notes: We have modified the capitalization issue of acid)**

| KEGG pathway | Top 5 up-regulated or down-regulated genes (Running ES) | | | | |
| --- | --- | --- | --- | --- | --- |
| Complement and coagulation cascades | Kng1 （0.034793712） | Procr（0.08890789） | LOC100911545（0.110159054） | F3（0.13385348） | F2（0.15698338） |
| IL-17 signaling pathway | Mmp13 (0.10663371) | Mmp3 (0.15979874) | Nfkbia (0.16893157) | Hsp90aa1 (0.19320148) | Ikbkb (0.20280871) |
| Primary bile acid biosynthesis | Baat (0.15700345) | Cyp27a1 (0.3128967) | Cyp39a1 (0.42751586) | Acox2 (0.51506096) | Hsd3b7 (0.60814) |
| Neuroactive ligand-receptor interaction | Adora2a (-0.31462246) | Gria3 (-0.31216073) | Ptger4 (-0.31295916) | Tacr3 (-0.3112585) | Thra (-0.3136276) |
| Fatty acid metabolism | Cpt1a (-0.3685738) | Elovl5 (-0.3621944) | RGD1560015 (-0.3497701) | Ehhadh (-0.34521574) | AABR07039037.1 (-0.34013155) |
| Axon guidance | Ntng1 (-0.43923038) | Arhgef12 (-0.43651527) | Fyn (-0.4378183) | LOC100910732 (-0.43523154) | Efna3 (-0.43854192) |

**Supplementary Figure 1**

**
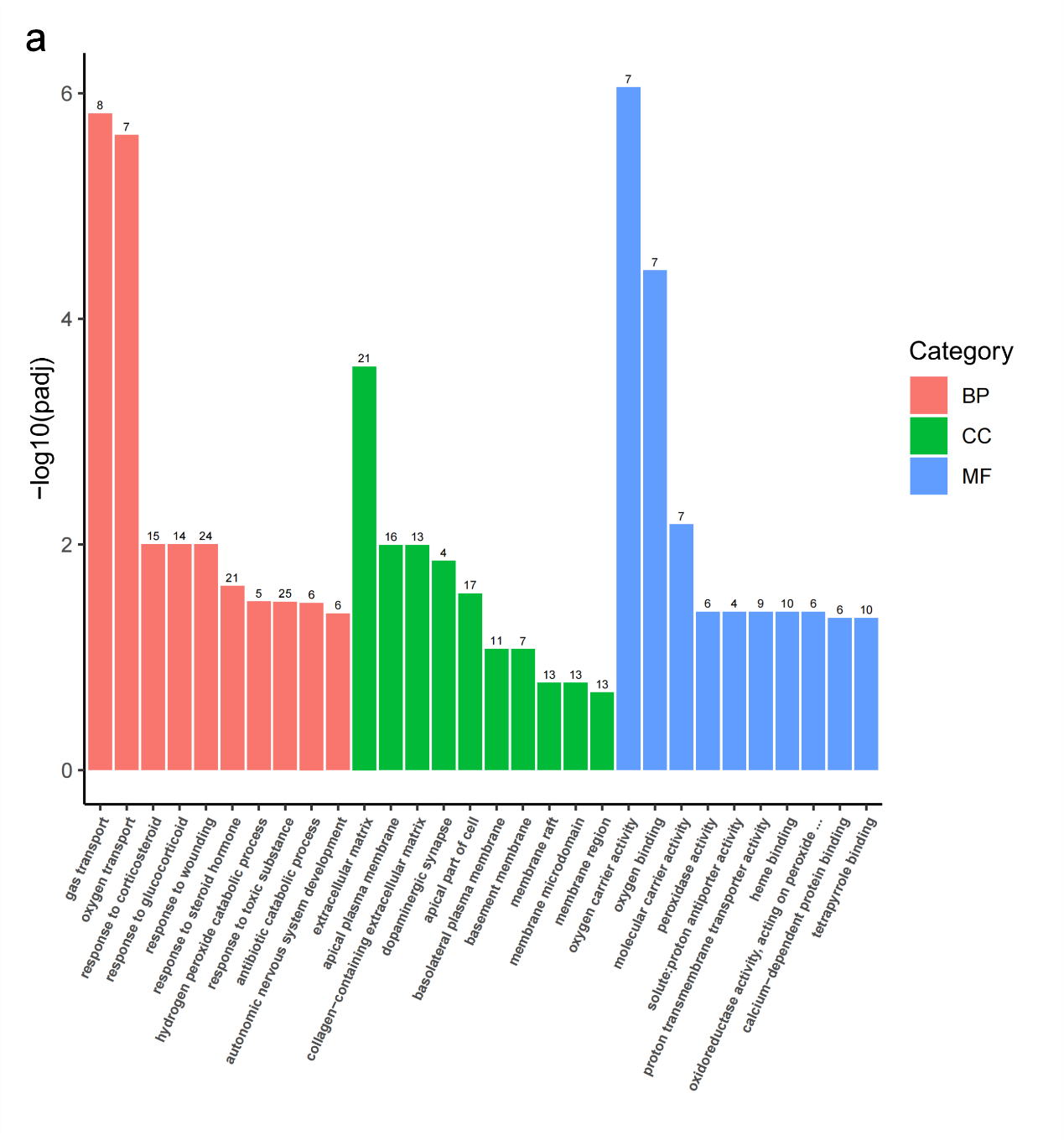
**

**Supplementary Figure 1 Functional analysis of all DEGs** a. Go enrichment analysis of DEGs. BP: Biological process; CC: Cellular component; MF: Molecular function.

**Supplemental Figure 2 (Notes: We have uploaded a clearer figure)**

**
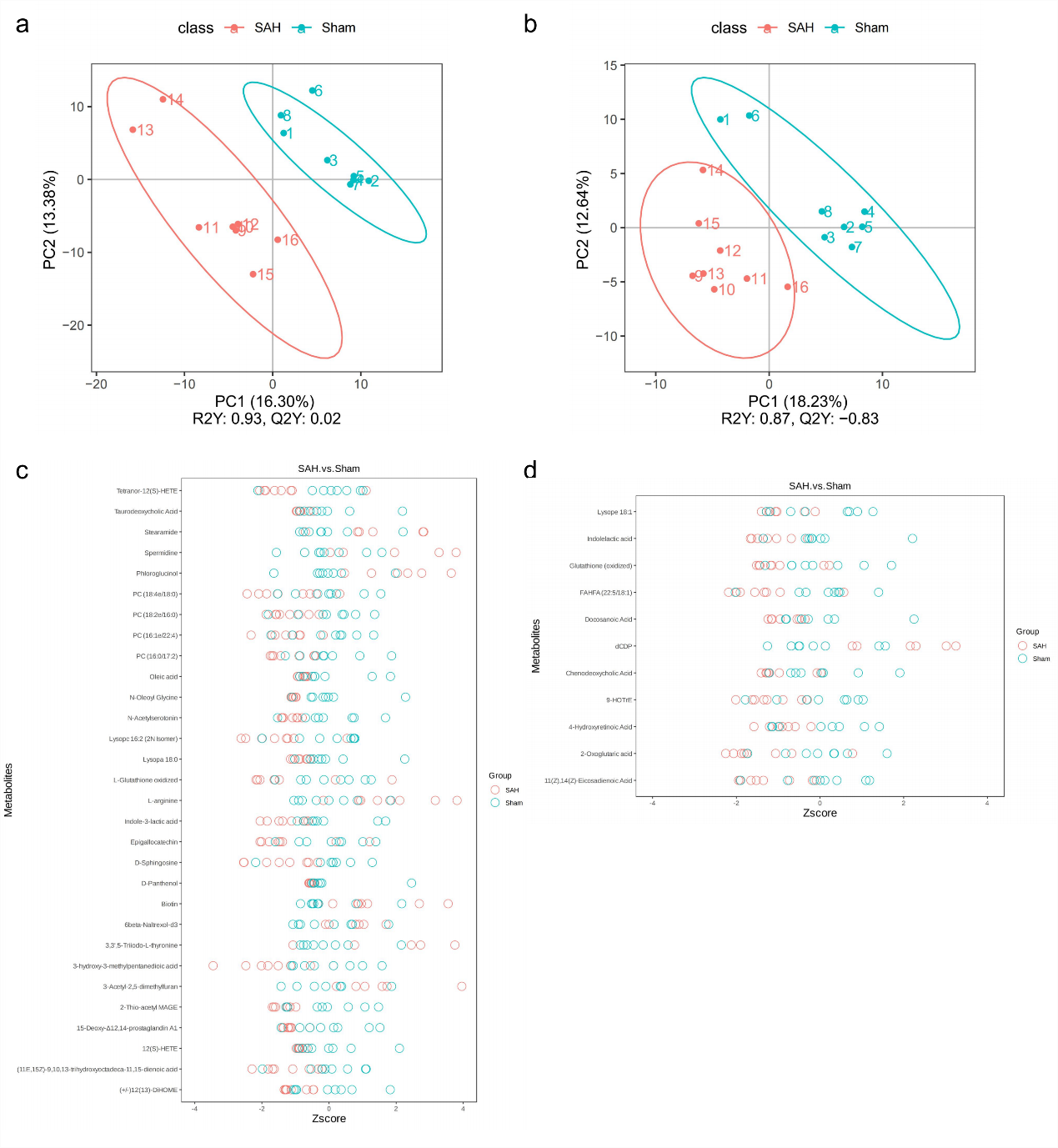
**

**Supplemental Figure 2 Data verification of PLS-DA and Z-score** PLS-DA in serum samples of SAH groups (red circle) and Sham groups (green circle) in the positive (a) and negative (b) ion model; Z-score plots of positive (c) and negative (d) ion model; red circle indicated SAH groups, while green circle indicated Sham groups.

**Supplemental Figure 3**

**
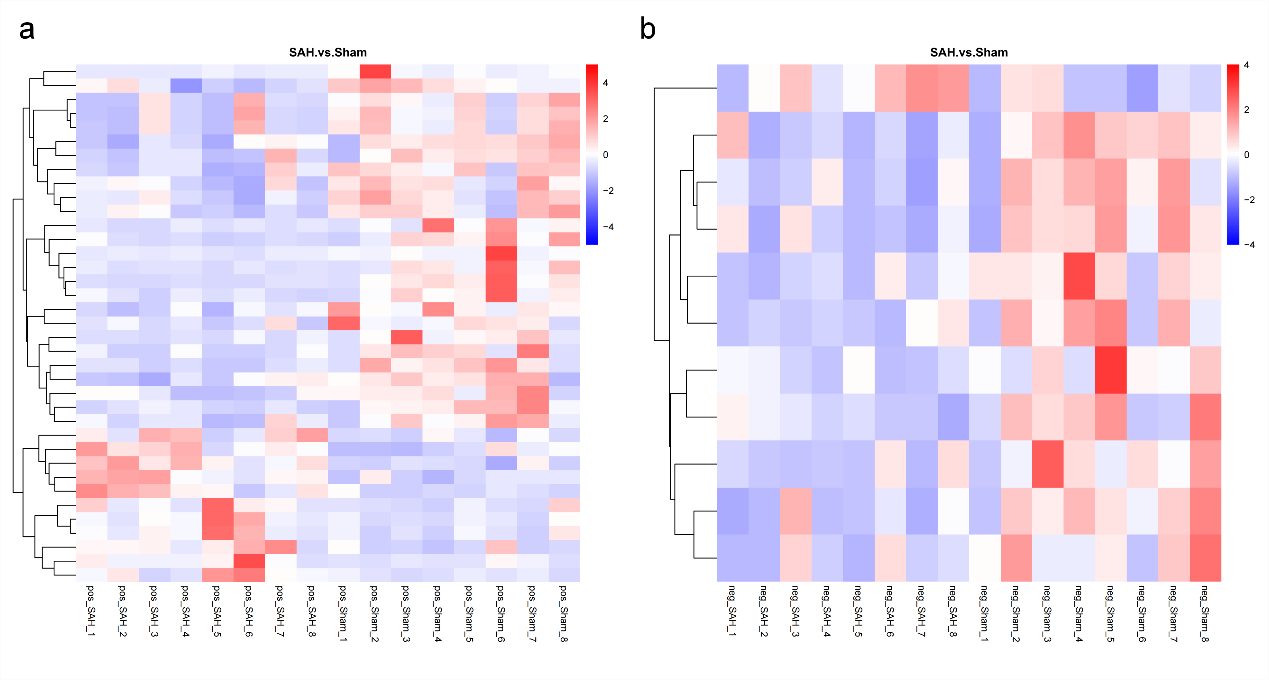
**

**Supplemental Figure 3 Cluster analysis of metabolites.** Heatmap showed cluster analysis of metabolites in positive ion model (a) and negative ion model (b).

**Supplementary Figure 4 (Notes: We have modified the numbering issue for Supplementary Figure 4, e and f)**

**
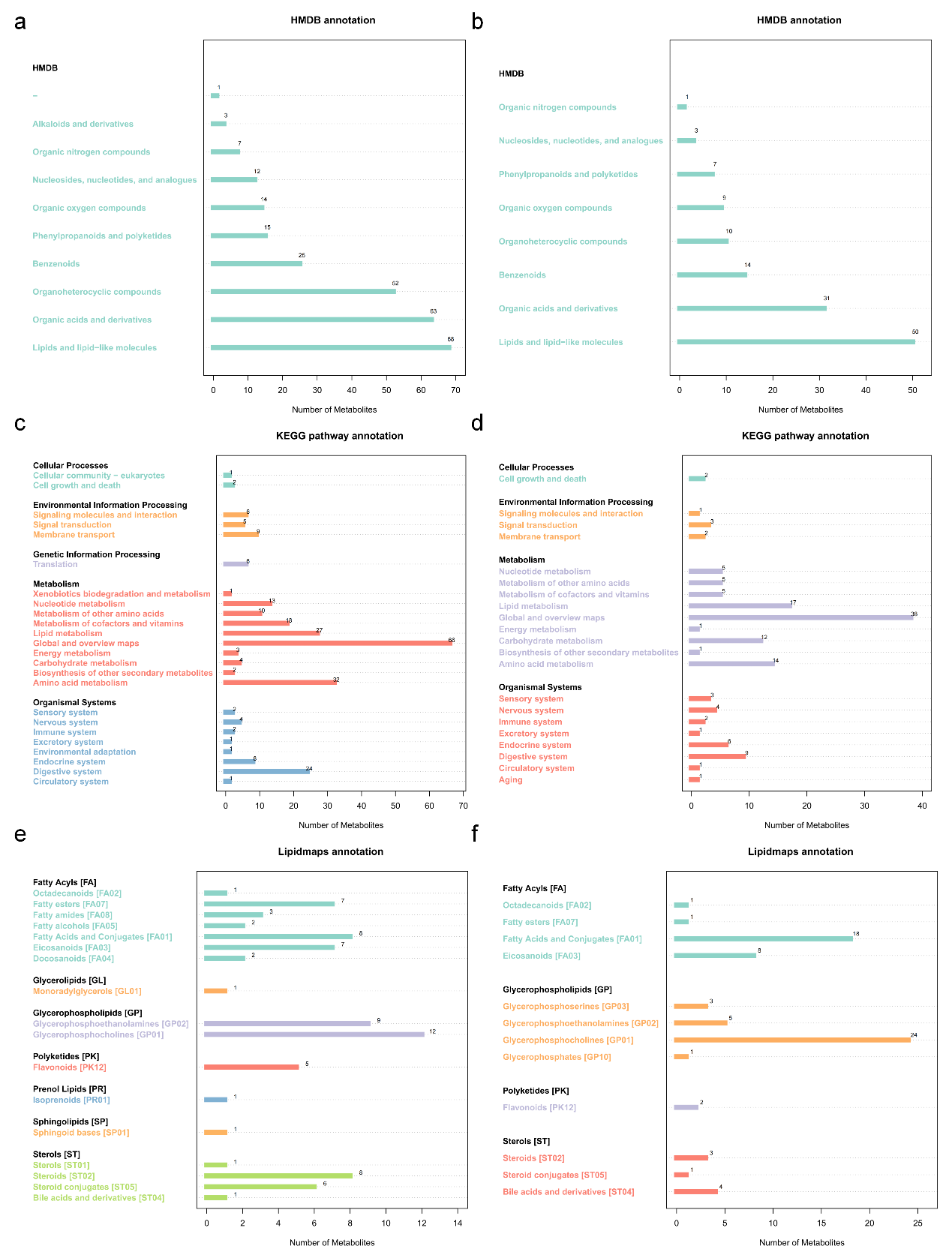
**

**Supplementary Figure 4 Functional and Taxonomic Annotation of Metabolites.** HMDB annotation in positive ion model (a) and negative ion model (b). KEGG annotation in positive ion model (c) and negative ion model (d). Lipidmaps annotation in positive ion model (e) and negative ion model (f).
